# Ectopic pancreatic pseudocyst and cyst presenting as a cervical and mediastinal mass - case report and review of the literature

**DOI:** 10.1186/1746-1596-8-176

**Published:** 2013-10-23

**Authors:** Ariel Rokach, Gabriel Izbicki, Maher Deeb, Naama Bogot, Nissim Arish, Irith Hadas-Halperen, Hava Azulai, Abraham Bohadana, Eli Golomb

**Affiliations:** 1 The Institute of Pulmonology, Shaare Zedek Medical Center, Jerusalem, Israel; 2 Department of Cardiothoracic Surgery, Shaare Zedek Medical Center, Jerusalem, Israel; 3 Department of Radiology, Shaare Zedek Medical Center, Jerusalem, Israel; 4 Institute of Pathology Shaare Zedek Medical Center, Jerusalem, 91031, Israel

**Keywords:** Ectopic pancreas, Mediastinum, Computed tomography, Pseudocyst, Cyst

## Abstract

Ectopic pancreas in the mediastinum is extremely rare. We are reporting on a case of a twenty two year old woman who presented to our clinic with a large cervical mass. The CT scan revealed a cystic lesion in the anterior mediastinum. The patient underwent surgical resection by cervical approach. A Cystic mass with pseudocysts, cysts and complete pancreatic tissue were found in pathology. There were no signs of pancreatitis or malignancy. No recurrence was observed after a follow up of four years. We reviewed the case reports describing this rare condition in the medical literature.

We conclude that the possibility of ectopic pancreatic tissue should be included in the differential diagnosis of anterior mediastinal cystic mass, though as a remote possibility. Surgery is probably needed for the diagnosis and treatment. Posterior mediastinal pseudocyst is a different entity associated with acute pancreatitis. In those cases surgery is not recommended. Our third conclusion is that pancreatic tissue should be actively sought, if a structure resembling a pseudocyst is found in an unexpected location.

**Virtual slides:**

The virtual slide(s) for this article can be found here: http://www.diagnosticpathology.diagnomx.eu/vs/1849369005957671

## Background

Congenital ectopic pancreas is a known phenomena found in the gastrointestinal tract in about 2% of autopsies [1]. An ectopic pancreas arising from the anterior mediastinum is extremely rare. To the best of our knowledge, only a few cases [2-19] have been reported in the literature, all of them arising from the anterior mediastinum. A different phenomenon are the pseudocysts arising from the posterior mediastinum as a result of acute pancreatitis [20-26]. Ectopic tissue arising from the mediastinum might contain other organs like spleen and endometrium.

The first reported case was published in 1957 by Shillitoe and Wilson [2]. Herein we describe a case report of asymptomatic ectopic pancreas in the anterior mediastinum. In our case report pseudocysts and cysts were the main pathologic findings. We reviewed the cases published in the literature and distinguished between ectopic pancreas arising from the anterior mediastinum and ectopic pancreas arising from the posterior mediastinum.

## Case presentation

**Figure 1:**
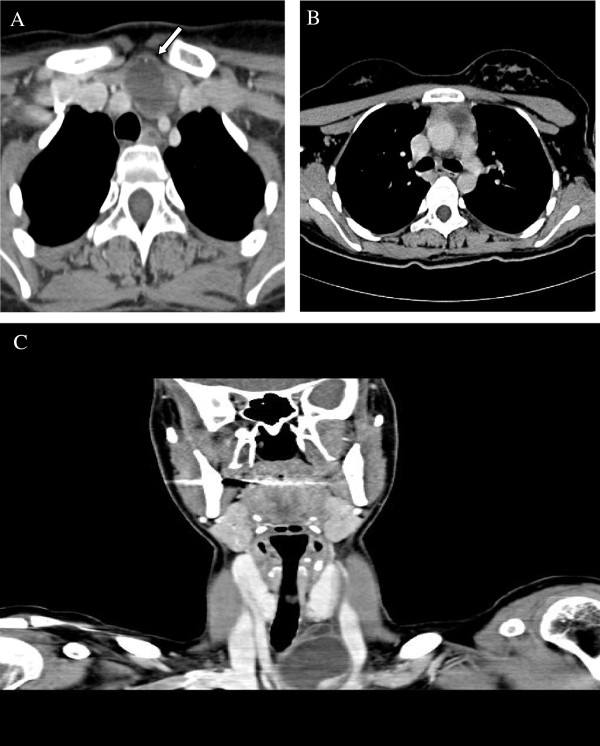
**Computed tomography images.** Axial Computed Tomography images at the level of the thoracic inlet, show two adjacent cystic lesions, one in the anterior lower neck **(A)** measuring 3.8×2.3×5.1 cm and one in the anterior mediastinum on the left **(B)**, measuring 2.7×2.2×1.8 cm. On coronal reconstruction **(C)** the neck lesion is shown as a lobular cystic mass with smooth enhancing rim. There is a speck of calcification at the cyst's wall (**A**, arrow).

**Figure 2:**
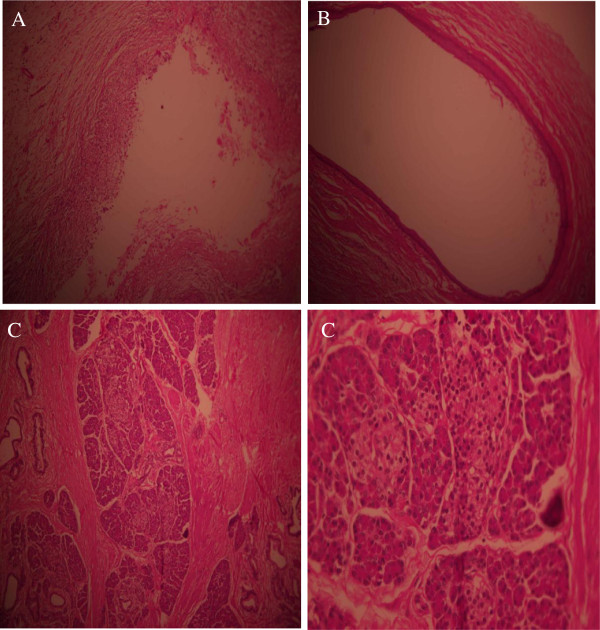
**Pathology slides.** Pathology slides show pseudo cyst **(A)**, cyst **(B)** and pancreatic tissue endocrine and exocrine pancreas **(C)**.

An otherwise healthy twenty two year old woman was sent to our hospital because of a left cervical mass. The mass was soft without tenderness and with no symptoms like chest pain, dyspnea or cough. The physical examination was unremarkable except the cervical mass. Blood tests including complete blood count, electrolytes and thyroid function tests were within the normal range. Cervical ultrasound revealed a left supraclvicular hypoechogenic lesion (2.4×3.8 cm). Chest computed tomography demonstrated a cystic mass in the anterior mediastinum protruding to the left cervical region, close to the left common carotid artery (Figure 1). Fine needle aspiration showed inflammatory cells without specific diagnosis and no signs of malignancy. Surgical resection was carried out by cervical approach. Pathology examination showed cystic masses along with pseudo cysts and a complete pancreatic tissue (including endocrine and exocrine tissue). There were no signs of malignancy (Figure 2). Surgical follow-up was uneventful. Four years after the operation the patient feels well with no signs of recurrence.

### Review of the literature

We performed a Pub med search in order to find all the published cases of ectopic pancreatic tissue in the Mediastinum. We reviewed the cases in order to describe this rare condition (Table 1).

**Table 1:** Ectopic pancreas in the anterior mediastinum- clinical features of 22 cases

**Reference**	**Gender**	**Age**	**Size (Cm)**	**Clinical presentation**	**Pathology**
Shillitoe [2] 1957	Female	15	5.5	Dyspnea, Night sweats	Benign
Carr [3] 1977	Female	57	10	None	Benign
Von Schweinitz [4] 1990	Male	5	5 × 5 × 5	Chronic Pneumonia	Benign
Perez-Ordonez [5] 1996	Female	16	12	None	Benign
Gong [6] 1997	Female	26	20 × 15	Chest pain, Cough	Benign
Gong [6] 1997	Female	26	4.3 × 1.3	Chest pain	Benign
Wu [7] 1998	Female	60	10 × 5	Chest pain	Benign
Cagirici [8] 2001	Female	45	10 × 8	Chest pain, cough	Benign
Sentis [9] 2004	Male	44	10 × 8 × 7.5	Chest pain, Dyspnea	Benign
Tamura [10] 2005	Male	39	10 × 8	Chest pain	Benign
Al-Salam [11] 2006	Male	40	8 × 6 × 6	Cervical swelling	Benign
Wang [12] 2007	Female	17	12 × 12 × 4	Chest pain, Dyspnea	Benign
Wang [12] 2007	Female	24	10 × 8 × 4	Chest pain, Dyspnea	Benign
Ehricht [13] 2009	Male	25	15 × 15	Pneumonia	Benign
Chen [14] 2009	Female	32	13 × 16 × 8	None	Benign
Fayoumi [15] 2010	Male	51	10 × 7 × 5	Chest pain, cough	Benign
Fayoumi [15] 2010	Male	42	10 × 5	Shoulder pain	Benign
Romain [16] 2011	Female	66	11 × 9	Chest pain	Malignant
Takemura [17] 2011	Female	21	3.5 × 3.5	Chest pain	Benign
Szabados [18] 2012	Male	32	4 × 4	Chest pain, Hemoptysis	Benign
Byun CS [19] 2012	Female	31	7 × 3 × 4	Chest pain, cough, Hemoptysis	Benign
Rokach	Female	22	5.1 × 3.8 × 2.3	None (Asymptomatic cervical mass)	Benign
Summery/Average	F-14	34	3.5-20	Sympomatic-18	Benign-21
	M-8			Asymptomatic-4	Malignant-1

In addition to our patient we found twenty one case reports of ectopic pancreas in the mediastinum. Fifteen case reports were written in English three in Chinese, two in German and one in Spanish. Most of the cases were described in young adults; Average age was thirty four and ages ranged from 5 to 66. The prevalence was higher in females (64% females). The clinical presentation was usually nonspecific including, chest pain, shoulder pain, shortness of breath, fever, neck swelling, night sweats, heart murmur, fatigue, chronic pulmonary infiltrate and tamponade. Four patients were asymptomatic at presentation. All the cases showed cystic lesions located in the anterior mediastinum. Most of the lesions were large ranging from 3.5 cm to 20 cm in diameter. Large lesions encompassed adjacent structures such as the great vessels. Associated findings were pleural effusions and pericardial effusions. There were no specific findings on the CT scan that could distinguish ectopic pancreatic tissue from other diagnosis. The cyst wall and the solid portion of the lesions usually showed mild to moderate enhancement with contrast material. The radiological appearance could not be distinguished from Thymoma, Lymphoma or Teratoma. In all cases the diagnosis was done only after surgery. Twenty one cases were benign and fully recovered after the operation and in a single case pancreatic carcinoma arising from the mediastinum was found. In the benign cases no recurrence was reported. The only patient with pancreatic adenocarcinoma died 15 months after the operation. This case strengthens the importance of surgery.

Ectopic pancreas in the mediastinum is a very rare condition. There are two theories on the embryogenesis of this anomalous development [4,8]. The first theory involves pluripotent epithelial cells of the ventral primary foregut underwent abnormal differentiation that led to the formation of ectopic pancreatic tissue in the anterior mediastinum. The second theory involves migration of cells from the pancreatic bud to a different site. Ectopic pancreas can be found in other locations as well. The most common site is the gastrointestinal tract. Pancreatic tissue is found there in two percent of autopsies.

A different entity is a pseudocyst arising from the posterior mediastinum as a result of acute pancreatitis [20-26]. In those cases extension of pancreatic necrosis into the mediastinum was possible via the aorta or the esophageal hiatus, through the diaphragmatic crura, or through erosion in the diaphragm.

Ectopic tissue in the mediastinum is a rare phenomenon. The anomalous development of ectopic tissue may occur due to abnormal differentiation of pluripotent cells, migration of ectopic cells or malignant transformation. A few case reports described this rare phenomenon. Hong Li et al. described a rare liposarcoma in the superior mediastinum [27]. The origin of this rare tumor was the Para pharyngeal region. Monika Saini et al. described intrapulmonary Teratoma attaching the medial mediastinum [28]. Composite lymphoma in the anterior mediastinum, a rare lymphoma from two different origins, was described by Guohua Yu et al. [29]. These cases represent malignant transformation. In our cases there were no signs of malignancy and the origin of the ectopic tissue was probably from abnormal differentiation of pluripotent cells or migration of ectopic cells.

## Conclusions

The first reported case of ectopic mediastinal pancreas was published in 1957 by Shillitoe and Wilson [2]. They described a fifteen year old female that had benign ectopic pancreatic tissue in the anterior mediastinum. Ectopic Pancreas in the anterior Mediastinum is extremely rare. Only twenty one case reports were described in the literature, all in young adults. The lesions were solid-cystic. The pathology and the clinical course were benign in twenty cases and malignant in one case. There were no signs of pancreatitis. Posterior mediastinal pseudocyst is a different entity associated with acute pancreatitis. In those cases surgery is not recommended.

We conclude that ectopic pancreas should be considered in the differential diagnosis of anterior mediastinal lesions. Surgery is probably needed for the diagnosis and treatment. Pancreatic tissue should be actively sought, if a structure that looks like a pseudocyst is found in an unexpected location.

## Consent

Written informed consent was obtained from the patient for the publication of this report and any accompanying images.
